# Consumption and acceptability of whole grain staples for lowering markers of diabetes risk among overweight and obese Tanzanian adults

**DOI:** 10.1186/1744-8603-9-26

**Published:** 2013-06-23

**Authors:** Alfa Muhihi, Dorothy Gimbi, Marina Njelekela, Emmanuel Shemaghembe, Kissah Mwambene, Faraja Chiwanga, Vasanti S Malik, Nicole M Wedick, Donna Spiegelman, Frank B Hu, Walter C Willett

**Affiliations:** 1 Clinical Trial Unit, Africa Academy for Public Health, Dar es Salaam, Tanzania; 2 Ifakara Health Institute, Ifakara, Morogoro, Tanzania; 3 Department of Food Sciences and Technology, Sokoine University of Agriculture, Morogoro, Tanzania; 4 Department of Physiology, Muhimbili University of Health and Allied Sciences, Dar es Salaam, Tanzania; 5 Department of Sociology and Anthropology, College of Arts and Social Sciences, University of Dar es Salaam, Dar es Salaam, Tanzania; 6 Department of Psychiatry and Mental Health, Muhimbili National Hospital, Dar es Salaam, Tanzania; 7 Department of Internal Medicine, Muhimbili National Hospital, Dar es Salaam, Tanzania; 8 Department of Nutrition, Harvard School of Public Health, Boston, MA, USA; 9 Department of Epidemiology, Harvard School of Public Health, Boston, MA, USA; 10 Department of Biostatistics, Harvard School of Public Health, Boston, MA, USA

**Keywords:** Acceptability, Brown rice, Unrefined carbohydrates, Obesity, Tanzania

## Abstract

**Background:**

Dietary changes characterized by a reduction in carbohydrate quality are occurring in developing countries and may be associated with a higher prevalence of obesity and chronic diseases such as type 2 diabetes mellitus. We assessed the preferences and acceptability of unrefined whole grain carbohydrate staples (i.e., brown rice, unrefined maize and unrefined sorghum ugali) as substitutes for commonly consumed refined carbohydrates in Tanzania.

**Methods:**

A questionnaire was used to collect sociodemographic information and dietary habits, and pre-and post-tasting questionnaires were administered for test foods. A 10-point LIKERT scale was used to rate attributes of the three test foods.

**Results:**

White rice and refined maize ugali were the most commonly consumed carbohydrate staples in this population; 98% and 91%, respectively. Occasional consumption of unrefined maize and sorghum ugali was reported by 32% and 23% of the participants, respectively. All of the test foods were highly rated for smell, taste, color, appearance and texture. Taste was rated highest for unrefined maize ugali. Almost all of the participants were willing to participate in a future dietary intervention involving regular consumption of these unrefined carbohydrates for at least six months duration.

**Conclusions:**

These findings suggest that whole grain carbohydrates are highly acceptable, and that there is a promising potential for their use in future dietary intervention studies in Tanzania.

## Background

Dietary habits are changing in both urban and rural areas of Tanzania. Traditionally, whole grain foods have been the basis of carbohydrate staples in Tanzania. However, white rice and refined maize ugali (stiff porridge prepared from refined maize flour), are now the most commonly consumed staple foods in urban areas [1] and among higher socio-economic groups [2]. Traditional unrefined ugali is still common in rural settings [1], although dietary transitions are also underway in these areas [3].

Consumption of refined carbohydrates such as white rice, have been strongly associated with development of type 2 diabetes mellitus (T2DM) [4]. Whole grain foods such as brown rice tend to have lower glycemic index values compared to refined grains [5], and have been associated with a decreased risk of T2DM [6-8]. As a consequence of urbanization and rapid nutrition transition, T2DM, which was once considered a disease of affluent societies, is now increasing in most developing countries [9], including those in sub-Saharan Africa, at unprecedented rates [10,11].

The International Diabetes Federation estimated that the prevalence of T2DM in Sub-Saharan Africa among people aged 20-70 years was 2.4% in 2003, with the majority of the burden carried by adults aged 40-59 years old [12]. The current estimated prevalence of diabetes in Sub-Saharan Africa is 3.2%, and is expected to rise to 3.7% by the year 2030 [13]. In Tanzania, the prevalence of T2DM was reported to be as low as 0.5% among rural inhabitants and 1.9% among urban dwellers three decades ago [14]. Since then, survey data indicate that the prevalence of T2DM in Tanzania has increased six-fold between 1989 and 2000 [15,16]. In 2003, there were approximately 380,000 people with T2DM in Tanzania, ranking it among the top five countries with the highest number of people affected by T2DM in Sub-Saharan Africa [17]. More recent estimates show that approximately half a million people have T2DM in Tanzania and that this number is expected to double by 2030 [13]. This increase in T2DM will result in substantial disease, disability and economic burdens, highlighting the importance of identifying simple and sustainable lifestyle interventions that can prevent the development of T2DM.

Substituting whole grain foods for refined grain staples may be an effective strategy to reduce risk of T2DM in populations undergoing nutrition transition. However, before doing so, assessing awareness, opinions and cultural acceptability of whole grains is imperative. We have previously shown that substituting brown rice for white rice is culturally acceptable and feasible in Shanghai, China [18] and Chennai India [19], where white rice consumption is also high. Therefore, in this study, we sought to assess the consumption and acceptability of brown rice, unrefined maize ugali (“dona”) and unrefined sorghum ugali (“mtama”) as whole grain substitutes for refined carbohydrate staples (white rice and refined maize ugali) in Tanzania. The findings from this study will be used to design future intervention trial to lower markers of T2DM risk in a similar urban Tanzanian population.

## Methods

### Study sites and participants

This study was conducted among 44 overweight and obese [body mass index (BMI) ≥ 25 kg/m^2^] adults in the Dar es Salaam, an urban region, and Morogoro, a semi-urban region, Tanzania. In Dar es Salaam, 23 participants were recruited from Muhimbili University of Health and Allied Sciences (MUHAS) and in Morogoro 21 participants were recruited from Sokoine University of Agriculture (SUA). Participants included nurses, office attendants, office secretaries, security officers and laboratory technicians.

The inclusion criteria for this study were: age between 40 and 65 years, BMI ≥ 25 kg/m^2^ and habitual consumption of rice and ugali defined as at least once per week. Posters were used at the two study sites to recruit participants. The study was approved by the Tanzania National Institute for Medical Research, and informed consent was obtained from all participants prior to conducting the study.

### Data collection procedures

On the first day, participants completed a self-administered questionnaire on sociodemographic information (age, sex, marital status, family size and education) and dietary habits, including their preferred carbohydrate staple foods. They also completed pre-tasting questionnaires pertaining to awareness, and market price of the test foods.

### Test foods and cooking methods

Brown rice, unrefined maize flour and unrefined sorghum flour were purchased from local vendors in Morogoro. To maintain consistency in preparation methods, test foods for both sites were cooked by the same nutritionist from SUA. Brown rice was cooked at a rice-to-water ratio of 1 to 2.5 using a charcoal cooker for approximately 45 to 60 minutes. Ugali was prepared using whole grain maize and sorghum flours, by adding small amounts of flour to cold water in a bowl and stirring well. The mixture was then poured into a pan of boiling water. While stirring continuously, flour was gradually added until the mixture was thickened into a stiff porridge. The overall flour-to-water ratio was approximately 1 to 5. Muhimbili National Hospital (Dar es Salaam) and Sokoine University of Agriculture (Morogoro) provided venues where the taste tests were conducted.

### Sensory analysis

The sensory analysis of the cooked test foods was conducted using descriptive analysis methods. The sensory attributes included smell, taste, color, appearance, texture, hardness and configuration. All the participants were trained on how to conduct the evaluation. Sensory evaluation was carried out in the following order; (a) Selection of an appropriate evaluation method suitable for descriptive analysis. (b) Training of participants on how to conduct sensor evaluation of the test food. (c) Lexicon of sensory attributes which was pre-determined by the study team by taking into consideration different quality characteristics of the cooked test foods. (d) Sensory evaluation by the study participants under standard conditions. (e) Data analysis by calculating the mean scores of the test foods on different sensory attributes.

Sensory evaluation was conducted for each type of test food per day (brown rice on Day 1, unrefined maize ugali on Day 2 and unrefined sorghum ugali on Day 3). Participants were served with an *ad libitum* meal of the test food for lunch with meat, beans, vegetables and water as the only beverage. Using descriptors and definitions (Lexicon) provided in Table 1, participants rated the intensity of sensory attributes on a 10 points LIKERT scale with ‘1’ representing “dislike very much” and ‘10’ representing “like very much”. The participants were asked to indicate the perceived intensity of each descriptor.

**Table 1:** **Lexicon of sensory attributes of the test foods** (**unrefined maize ugali**, **brown rice and unrefined sorghum ugali**)

**Term**	**Definition**
Smell	The perceived scent of the test food during eating
Taste	The general term used to describe the sweet, bitter, sour or salty taste of the test food on the tongue
Appearance	The outwards aspect of the test food
Color	Degree of evenness of the color of the test food
Texture	Smoothness or roughness of a chewed mass of the taste food
Shine	The degree of brightness of the test food
Hardness	Reflected by the amount of energy/force required to compress a sample of food between the tongue and the palate
Configuration	The external form or outline of the shape of the test food

The preferred times (breakfast, lunch, dinner or in-between meals) and approximate amount of consumption of staple carbohydrate foods were also ascertained. We used a measured sample plate of cooked food (660 grams for each staple considered) as a reference, and asked participants to estimate the amount rice and ugali they usually consumed during the respective meals. Estimated amounts were compared to the quantity of the reference (i.e., half, the same, 1.5 and 2.5 times).

### Data analysis

The questionnaire data were reviewed and entered into an Excel spreadsheet before being imported into Statistical Analysis Software version 9.2 (SAS Institute Inc., North Carolina, USA) for analysis. Sociodemographic characteristics of the study population were described using standard descriptive statistics such as means, standard deviations, and frequencies. Differences by gender and region were assessed using *X*^*2*^ tests for categorical variables and Wilcoxon tests for continuous variables. Differences in the ratings of the three taste foods; brown rice, unrefined maize ugali and unrefined sorghum ugali were assessed using a non-parametric Kruskal-Wallis test. In all analyses, a two-tailed p-value ≤ 0.05 was considered statistically significant.

## Results

### Description of the study participants

Characteristics of the study population are presented in Table 2. Mean age and BMI of the study participants were 48±6 years (range 40-60) and 32±5 kg/m^2^ (range 25.2-45.4), respectively. The average BMI of women was higher than that of men (p = 0.03). Gender differences were also observed for level and years of education, with fewer women having attained advanced education (p < 0.05). Significant differences according to study region were observed for education level with more participants from Morogoro having attained advanced education compared to their counterparts in Dar es Salaam (p < 0.001). Both white rice and refined maize flour were priced significantly higher in Dar es Salaam compared to Morogoro (white rice, p-value=0.05; refined maize flour, p-value < 0.001).

**Table 2:** **Sociodemographic characteristics of the study population by gender and region** (**N**=**46**)

	**Gender**		p - value*	**Region**		p - value*
	**Men(N =** **18)**	**Women(N =** **26)**		**Dar es Salaam(N =** **23)**	**Morogoro(N =** **21)**	
**Age(years)**	48 ± 5	48 ± 5	0.77	48 ± 6	47 ± 5	0.46
**Body mass index(kg/m**^**2**^**)**	30 ± 5	33 ± 5	0.03	31 ± 4	33 ± 5	0.39
**Marital status (%)**						
Single	0	16		-	19	
Married	89	65	0.14	83	67	0.09
Other	11	19		17	14	
**Education level(%)**						
Secondary	33	46		70	9	
Vocational	23	50	0.01	17	62	<0.001
Advanced secondary	11	0		4	5	
College/University	33	4		9	24	
Household size**						
Total	-	-	-	7 ± 3	6 ± 2	0.29
Adults (≥ 18 years)	-	-	-	4 ± 2	4 ± 1	0.39
Children (< 18 years)	-	-	-	3 ± 2	3 ± 1	0.76
Price/Kg**(Tshs)^**†**^						
White rice	-	-	-	1300 ± 300	1100 ± 340	0.05
Refined maize flour	-	-	-	770 ± 80	660 ± 80	<0.001

*P-values are presented as X^2^ tests for categorical variables and Wilcoxon for continuous variables.

**Family size and price/kg of white rice and refined maize flour are not expected to differ by gender.

^†^1 US dollar = 1585 Tanzanian shillings (Tshs).

### Preference and frequency of consumption of selected carbohydrate staples

White rice and refined maize ugali were the most preferred and commonly consumed carbohydrate staples (Table 3). About one-third of the participants reported that they consumed unrefined maize ugali at least a few times per month. At least one-fifth of the participants reported consumption of unrefined sorghum ugali, whereas only 11.4% had ever tried brown rice. There was no difference in the frequency of consumption of the above foods by either gender or region (results not shown). Other foods such as cooked green bananas, sweet and Irish potatoes, cassava, yams and a mixture of maize and beans (“makande”) were also popular in this population.

**Table 3:** Preference and frequency of consumption of some selected carbohydrate staples

**Overall preference (%)**					
**Food**	**1**^**st**^**choice**	**2**^**nd**^**choice**		**3**^**rd**^**choice**	**Other choices**
White rice	61	34		-	5
Refined maize ugali	34	55		9	2
Banana	5	5		34	56
Sweet potatoes	-	2		7	91
Irish potatoes	-	2		21	77
Cassava	-	-		7	93
Yams	-	-		2	98
Beans and maize mixed	-	-		9	91
**Frequency of consumption (%)**					
	**Rice**		**Ugali**		
**Frequency of consumption**	**White rice(N=44)**	**Brown rice(N=5)**	**Refined maize ugali(N=44)**	**Unrefined maize ugali(N=43)**	**Unrefined sorghum ugali(N=10)**
Tried once/few times a year	0	40	0	67	50
Few times/month	2	20	9	14	40
1-4 times/week	27	0	23	14	0
5-7 times/week	64	20	59	5	10
>Once/day	7	20	9	0	0

### Preferred time of consumption and quantity of the selected carbohydrate staples

Lunch and dinner were the preferred times for consumption of rice and ugali. Refined maize ugali was consumed preferably during lunch and white rice during dinner (data not shown). Most participants consumed approximately the same quantity of staple foods as the reference used in our study (i.e., 660 grams of cooked brown rice and 600 grams of cooked unrefined maize and sorghum ugali); white rice (55%), refined maize ugali (52%), unrefined maize ugali (51%) and unrefined sorghum ugali (44%). At least one-third of the participants consumed amounts exceeding the reference; white rice (39%) refined maize ugali (41%), unrefined maize ugali (38%) and unrefined sorghum ugali (33%).

### Factors influencing food choices

Table 4 shows the factors influencing food choices. Customs/tradition was the most important factor reported, influencing choice of food differently by both gender and region (p < 0.05). Taste and cost had borderline significant results by gender and region (p = 0.06 and p = 0.07, respectively). There were no statistically significant differences by gender or region for ease of preparation, availability and health reasons.

**Table 4:** Factors influencing choice of foods by gender and region

**Factors**	**All**	**Gender (%)**		**P - value**	**Region (%)**		**P -** **value**
		**Men**	**Women**		**Dar es Salaam**	**Morogoro**	
Cost	38	44	35	0.51	26	52	0.07
Taste	34	50	23	0.06	26	43	0.24
Health reasons	36	22	46	0.10	39	33	0.69
Easy Preparation	21	28	15	0.31	17	24	0.60
Availability	46	39	50	0.47	35	57	0.14
Customs/traditions	41	61	27	0.02	26	57	0.04

### Post-tasting assessment of brown rice, unrefined maize and unrefined sorghum ugali

The mean scores of ratings for brown rice, unrefined maize ugali and unrefined sorghum ugali according to smell, taste, color, appearance, texture, hardness, shine and configuration are provided in Table 5. Overall, there were no significant differences reported for any of the aforementioned properties between the three foods tasted, except for taste of unrefined maize and sorghum ugali, which was rated higher than brown rice (p < 0.01). Women rated the taste of unrefined maize and sorghum ugali significantly higher than brown rice (p-value < 0.01), whereas no significant differences were observed for men. Among participants from Dar es Salaam, taste and appearance were scored higher for unrefined maize ugali, and texture was scored lower for brown rice compared to unrefined maize and sorghum ugali. In Morogoro, smell and configuration were rated significantly lower for brown rice.

**Table 5:** **Mean scores of post**-**taste rating of the food by gender and region** (**Out of 10 points**)

	**Overall rating (Mean±SD)**							**P - value**
**Rating parameter**		**Brown rice**		**Unrefined maize ugali**		**Unrefined sorghum ugali**		
Smell		7 ± 3		8 ± 3		7 ± 2		0.63
Taste		7 ± 2		8 ± 2		8 ± 3		<0.01
Appearance		7 ± 2		7 ± 3		6 ± 3		0.21
Color		7 ± 3		7 ± 2		6 ± 3		0.21
Texture		8 ± 2		8 ± 3		9 ± 2		0.06
Shine		6 ± 2		7 ± 3		6 ± 3		0.50
Hardness		7 ± 2		8 ± 3		8 ± 2		0.06
Configuration		7 ± 2		8 ± 2		7 ± 2		0.10
**Rating by gender (Mean±SD)**								
**Rating parameter**	**Men (N = 18)**			**p - value**	**Women (N = 26)**			**p - value**
	**Brown rice**	**Unrefined maize ugali**	**Unrefined sorghum ugali**		**Brown rice**	**Unrefined maize ugali**	**Unrefined sorghum ugali**	
Smell	7 ± 3	7 ± 3	6 ± 2	0.26	7 ± 2	8 ± 2	8 ± 2	0.33
Taste	6 ± 3	8 ± 2	7 ± 3	0.14	7 ± 2	8 ± 2	8 ± 3	<0.01
Appearance	7 ± 2	7 ± 3	6 ± 3	0.12	7 ± 2	7 ± 2	7 ± 3	0.80
Color	6 ± 3	7 ± 3	6 ± 3	0.46	7 ± 2	7 ± 2	6 ± 2	0.45
Texture	7 ± 3	8 ± 2	8 ± 2	0.51	8 ± 2	9 ± 3	9 ± 2	0.06
Hardness	7 ± 2	7 ± 3	8 ± 3	0.62	8 ± 2	8 ± 3	9 ± 2	0.06
Shine	6 ± 2	7 ± 3	6 ± 3	0.17	7 ± 2	7 ± 2	7 ± 3	1.00
Configuration	7 ± 2	7 ± 2	7 ± 2	0.79	7 ± 2	8 ± 2	7 ± 2	0.08
**Rating by region (Mean±SD)**								
**Rating parameter**	**Dar es Salaam (N = 23)**			**p - value**	**Morogoro (N = 21)**			**p - value**
	**Brown rice**	**Unrefined maize ugali**	**Unrefined sorghum ugali**		**Brown rice**	**Unrefined maize ugali**	**Unrefined sorghum ugali**	
Smell	8 ± 2	8 ± 3	7 ± 2	0.19	6 ± 2	7 ± 3	7 ± 2	0.04
Taste	7 ± 3	9 ± 2	7 ± 3	0.02	7 ± 2	8 ± 2	8 ± 2	0.14
Appearance	7 ± 2	8 ± 3	6 ± 3	0.05	6 ± 2	7 ± 2	7 ± 2	0.48
Color	6 ± 3	8 ± 2	7 ± 3	0.25	7 ± 2	7 ± 2	6 ± 2	0.26
Texture	7 ± 3	9 ± 3	9 ± 2	0.01	8 ± 2	8 ± 3	8 ± 2	0.92
Hardness	7 ± 2	8 ± 3	9 ± 2	0.08	6 ± 2	7 ± 3	8 ± 2	0.50
Shine	7 ± 3	7 ± 3	7 ± 3	0.48	6 ± 2	6 ± 2	6 ± 2	0.60
Configuration	7 ± 3	8 ± 3	7 ± 3	0.69	7 ± 2	8 ± 2	8 ± 2	0.04

### Willingness to participate in a future dietary intervention study

All participants expressed willingness to participate in a future dietary intervention involving daily consumption of brown rice during lunch. At least 98% were willing to participate in an intervention involving consumption of either unrefined maize ugali or unrefined sorghum ugali. Nearly all participants were willing to participate in the study for at least six months (brown rice 95%, unrefined maize ugali 98% and unrefined sorghum ugali 95%).

## Discussion

This pilot study was conducted to assess the consumption and acceptability of whole grain staple foods as a first step in the design of a dietary intervention aiming at lowering markers of T2DM risk among adults in Tanzania. Overall, brown rice, unrefined maize and sorghum ugali were rated favorably in terms of smell, taste, color, appearance, texture, hardness, shine and configuration. Findings from this pilot study demonstrate the feasibility of a longer-term dietary intervention involving consumption of the selected whole grain foods.

White rice and refined maize ugali are currently the most frequently consumed carbohydrates in this population. With the burden of T2DM increasing at a greater rate in urban areas [15,16], dietary interventions targeting a shift back to traditional whole grain diets may have a major effect in reducing the incidence of DM among adult urban Tanzanians.

Observational studies have found that higher intakes of whole grains and dietary fiber are associated with a reduced risk of T2DM [7,8], metabolic syndrome and other diet-related chronic diseases [20,21]. In contrast, refined carbohydrates such as refined maize and white rice, have been strongly associated with the development of T2DM [4,22]. The main difference between whole and refined grains lies in the preservation of the bran and germ layers in whole grains, which are removed during processing and refinement [23]. Fiber which is concentrated in the bran fraction delays gastric emptying and hence leads to slow release of glucose into circulation [24,25]. Whole grains also have a higher content of essential fatty acids and various micronutrients such as magnesium, which may also contribute to the observed inverse association between whole grains and DM [26]. Benefits of whole grains on DM risk are hypothesized to be mediated through improved glycemic control, improved insulin sensitivity, reduced inflammation and favorable lipid profiles [23].

Traditions and customs played a significant role in influencing food choice among residents of Morogoro region, but not in Dar es Salaam, indicating that people from less urbanized settings of Tanzania still value their traditional diets. Consumption of whole grains, such as unrefined maize ugali in Tanzania, has been linked to low socioeconomic status, with persons eating unrefined maize ugali being regarded as poor [27]. As a consequence, majority of people, especially in urban settings, have shifted from consumption of unrefined to refined maize ugali. These findings are supported by another study conducted in Dar es Salaam which showed that white rice intake increases with increasing level of income [2].

Brown rice, unrefined maize ugali and unrefined sorghum ugali were all rated positively by the participants, indicating that they were acceptable, palatable and well-tolerated. Brown rice is not very common among Tanzanians and only five participants (four from Morogoro and one from Dar es Salaam) reported ever having tried brown rice before. Despite the fact that the majority of participants were tasting brown rice for the first time, responses were generally favorable. It is important to note that nearly all participants were willing to participate in a future dietary intervention study involving the consumption of brown rice, unrefined maize ugali or unrefined sorghum ugali to lower markers of DM risk. Similar findings by our group have been shown in Shanghai China [18] and Chennai, India [19], indicating that whole grain substitution of refined grain carbohydrate staples may be a culturally acceptable and feasible intervention strategy to reduce risk of diabetes in high-risk populations in many places around the world.

The main limitation of the current pilot study is its small sample size, which may have resulted in insufficient power to detect gender and regional differences in the acceptability of the unrefined staple dishes. Our analysis also lacks positive control which would have been indeed useful to get a clear picture by having both unrefined and refined carbohydrate foods tested and rated by the participants. While the selected study population is likely to be representative of those at higher risk of DM, it is possible that findings may not be generalizable to the broader Tanzanian population. However, by including the two diverse geographical regions, we were at least able to evaluate people from different socioeconomic and cultural backgrounds.

## Conclusions

These findings indicate that despite their currently low consumption rate, unrefined carbohydrate staples are highly acceptable in this population. As the majority of participants were willing to participate in a future intervention study involving consumption of brown rice, unrefined maize or sorghum ugali, there is an excellent potential for their use as intervention foods in future studies in Tanzania, leading to evidence-based public health nutrition programs aimed at mitigating the looming Tanzanian diabetes epidemic. Such evidence will also guide public health initiatives to raise awareness of the nutritional benefits of replacing refined carbohydrate staples with whole grain alternatives.

## Competing interests

The authors declare that they have no competing interests.

## Authors’ contributions

AM participated in data collection, analyzed and interpreted the data, drafted and revised the manuscript. DG participated in data collection data and critically reviewed the manuscript. MN conceived and designed the study, participated in data collection, critically reviewed the manuscript. ES moderated the focus group discussion and reviewed the manuscript. KM and FC participated in data collection and reviewed the manuscript. VSM, NMW, DS, FBH and WCW designed the study and critically reviewed the manuscript. All authors read and approved the final manuscript version.
